# Quantification of cell death and proliferation of patient-derived ovarian cancer organoids through 3D imaging and image analysis

**DOI:** 10.1016/j.xpro.2023.102683

**Published:** 2023-11-16

**Authors:** Aikaterini Skorda, Anna Røssberg Lauridsen, Kaisa Huhtinen, Alexandra Lahtinen, Wojciech Senkowski, Jaana Oikkonen, Johanna Hynninen, Sampsa Hautaniemi, Tuula Kallunki

**Affiliations:** 1 Danish Cancer Institute, Danish Cancer Society, 2100 Copenhagen, Denmark; 2 Research Program in Systems Oncology, Research Programs Unit, Faculty of Medicine, University of Helsinki, 00014 Helsinki, Finland; 3 Institute of Biomedicine and FICAN West Cancer Centre, University of Turku and Turku University Hospital, 20014 Turku, Finland; 4 Biotech Research and Innovation Centre, University of Copenhagen, 2200 Copenhagen, Denmark; 5 Department of Obstetrics and Gynaecology, University of Turku and Turku University Hospital, 200521 Turku, Finland; 6 Drug Design and Pharmacology, University of Copenhagen, 2200 Copenhagen, Denmark

**Keywords:** Cell Biology, Cancer, High-Throughput Screening, Organoids

## Abstract

Patient-derived organoids (PDOs) are ideal *ex vivo* model systems to study cancer progression and drug resistance mechanisms. Here, we present a protocol for measuring drug efficacy in three-dimensional (3D) high-grade serous ovarian cancer PDO cultures through quantification of cytotoxicity using propidium iodide incorporation in dead cells. We also provide detailed steps to analyze proliferation of PDOs using the Ki67 biomarker. We describe steps for sample processing, immunofluorescent staining, high-throughput confocal imaging, and image-based quantification for 3D cultures.

For complete details on the use and execution of this protocol, please refer to Lahtinen et al. (2023).^1^

## Highlights

• High-throughput workflows to evaluate the efficacy of cancer drugs in HGSC organoids
• PI staining-based cell death assay for 3D tumor organoids
• Steps to quantify and identify proliferative cancer cells via Ki67-based 3D imaging

## Abstract (editor highlights)

Publisher’s note: Undertaking any experimental protocol requires adherence to local institutional guidelines for laboratory safety and ethics.

## Graphical abstract

**Figure:**
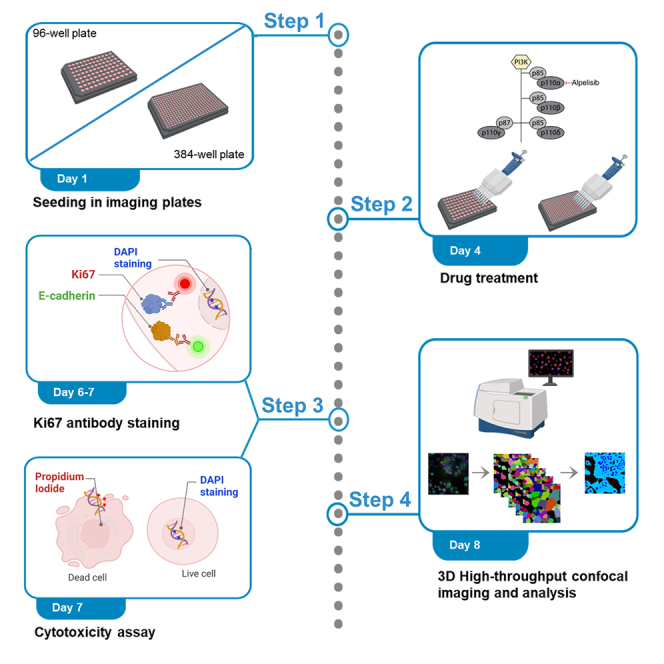

## Before you begin

Epithelial ovarian cancer (OC) research needs physiologically and pathologically relevant platforms to depict the disease heterogeneity and allow personalized medicine approaches to identify alternative treatments. While the establishment of OC patient-derived tumor organoids (PDOs) is getting more robust, the setup of basic biological assays and especially 3-dimensional (3D) high-content immunofluorescent microscopy is under development.^2^^,^^3^^,^^4^^,^^5^^,^^6^^,^^7^^,^^8^

Patient tumor-derived cancer cells, grown as self-renewable 3D organoid cultures, faithfully recapitulate their parental tumors *ex vivo* as they retain the genetic heterogeneity and the morphological structure of the original tumor.^9^^,^^10^ Exemplifying higher translational accuracy than the cancer cell lines grown in 2-dimensional (2D) cultures,^11^^,^^12^^,^^13^ PDOs are ideal *ex vivo* model systems to study cancer progression and drug resistance mechanisms, but also to screen for efficient drug treatments and possibly, guide clinical decisions.^14^

This dual protocol presents two recently established key application methods to evaluate drug effect, in terms of cell cytotoxicity and proliferation of high-grade serous ovarian cancer (HGSC) PDOs with an unbiased, automated high-throughput microscopy and image analysis.^1^ To measure cytotoxicity of PDOs perform Staining 1 from step 22 or to detect and quantify proliferation perform Staining 2 from step 33.

In our study, we combined bioinformatics with basic biology. PDOs and drug treatments were chosen based on tumor evolution findings connected to phosphoinositide 3-kinase/ Protein kinase B (PI3K/Akt) enhanced activity with worse HGSC survival. PDOs with genetic profile that fits the criteria and drugs targeting the pathway were chosen. However, these methods may be employed with PDOs derived from different cancer tissue and different drug treatments. Additionally, the staining conditions also apply for other proteins of interest; these include but are not limited to acetylated tubulin, CD133, p21 and cytokeratin 7/8.

This study is a part of DECIDER trial, ClinicalTrials.gov Identifier: NCT04846933, accessed at https://clinicaltrials.gov/ct2/show/NCT04846933.

### Institutional permissions

Tumor tissues used to generate the data presented in this protocol were obtained with the informed consent of the patients during routine surgery or laparoscopy. The study was approved by the Ethics Committee of Southwest Finland (statement number ETMK: 145 /1801/2015 § 585).

### Patient-derived organoid establishment and culture

**Figure 1:**
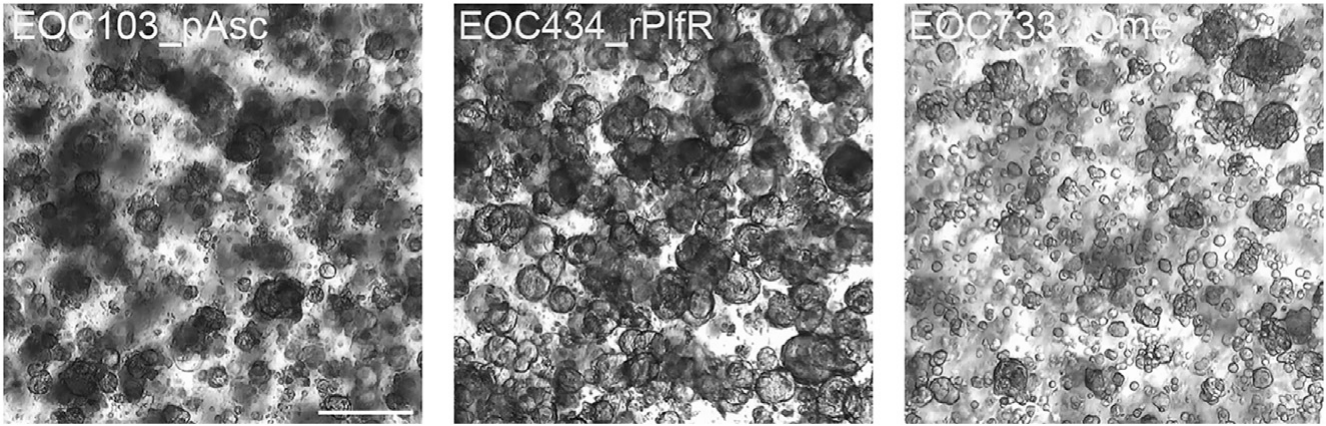
HGSC PDOs from different annotated patients and site of tumor biopsy These images show PDOs cultured in BME-2 and reaching appropriate size and confluency to be used for functional assays. The images show PDOs between passages 10 and 20, when they are used for assaying. Tissue abbreviations: p, primary; i, interval; r, recurrent; Asc, ascites; Plf, peritoneal lavage fluid; Ome, omentum. Scale bar is 100 μm.

HGSC PDOs are established and cultured as published by Senkowski et al.^15^ PDOs are cultured by embedding them in Reduced Growth Factor Basement Membrane Extract, Type 2 (BME-2) domes (droplets) attached at the bottom of a 6-well cell culture plate while submerged with indicated growth media.^1^ PDOs are grown until every droplet in the same well has reached approximately 90% confluency. For examples of PDOs that are ready for processing, see Figure 1. Tumor organoids established from other type of tumors will require growth media that they have been adapted to those.

However, in the experiments the growth media of organoids is changed to PDO experimental media after seeding for 3–4 days before drug treatment (see below in materials and methods). This media is selected due to its resemblance to human plasma, which has been shown to contribute to drug metabolism.^16^

### BME-2 preparation

**Timing: 15 min working time after 12–18 h thawing**

**CRITICAL:** Correct and careful thawing of the BME-2 is critical since inappropriately processed BME-2 stocks may lead to the failure of embedding of the tumorigenic cells and subsequent failure of organoid growth.

1. Pre-thaw a fresh aliquot of 5 mL stock of BME-2 in a bucket filled with ice and closed lid at 4°C for approximately 12–18 h. BME-2 must thaw gradually to avoid collapse of its structure and components.

***Note:*** Avoid multiple freeze-thaw cycles of BME-2 aliquots. We recommend using an aliquot maximum of two times.

***Note:*** From this step onwards, work under sterile conditions and consistently keep reagents and tools on ice. When handling the BME-2, always pre-cool the pipette tips prior to use by pipetting cold and sterile phosphate buffered saline (PBS).

2. Always begin with BME-2 protein concentration of 7.5 mg/mL. Since every batch usually varies, adjust the concentration by diluting with cold PBS accordingly.
3. Transfer the amount of BME-2 that is needed for the seeding in pre-cooled tubes on ice.
4. Dilute the BME-2 further with cold PBS according to the protocols below.

***Note:*** Mix the BME-2 dilution by pipetting up and down gently and slowly avoiding the creation of air bubbles.

***Note:*** Calculate in advance the amount of the BME-2 to be used and dilute only needed amount with PBS. Remember to prepare 10% extra dilution for pipetting. We do not recommend freezing the remaining of this BME-2 with PBS solution.

## Key resources table

**Table:** 

REAGENT or RESOURCE	SOURCE	IDENTIFIER
**Antibodies**		

Mouse anti-Ki67 antigen, clone MM1 (monoclonal) (1:150)	Monsanto	Cat# MONX10283
Donkey anti-mouse IgG (H + L), Alexa Fluor 594 (1:1,000)	Thermo Fisher Scientific	Cat# A21203
E-cadherin (ab11512), rat mAb [DECMA-1] (1:250)	Abcam	Cat# ab11512
Goat anti-rat IgG (H + L) secondary antibody, Alexa Fluor 488 (1:1,000)	Invitrogen	Cat# A-11006

**Biological samples**		

Ovarian cancer tissue and ascites	Turku University Hospital	NA

**Chemicals, peptides, and recombinant proteins**		

Plasmax	Ximbio	Cat# 156371
Human plasma-like medium (HPLM)	Gibco	Cat# A4899101
Phosphate-buffered saline (PBS)	Gibco	Cat# 10010023
TrypLE Express	Gibco	Cat# 12604013
HEPES, pH 7.4	Homemade	N/A
2-Hydroxybutyric acid	Sigma-Aldrich	Cat# 220116-5G
Primocin	InvivoGen	Cat# ant-pm-1
B-27 supplement (50X), minus vitamin A	Thermo Fisher Scientific	Cat# 12587010
p38/SAPK2 inhibitor (SB202190)	MedChemExpress	Cat# HY-10295
A-83-01	Sigma-Aldrich	Cat# SML0788
β-Estradiol	Merck	Cat# E2758
Recombinant human FGF-10	PeproTech	Cat# 100-26
Recombinant human FGF-4	PeproTech	Cat# 100-31
Recombinant human heregulinβ-1	PeproTech	Cat# 100-03
Animal-free recombinant human EGF	PeproTech	Cat# AF-100-15
Hydrocortisone	Sigma-Aldrich	Cat# H0888
Forskolin	MedChemExpress	Cat# HY-15371
Y-27632	MedChemExpress	Cat# HY-10583
Propidium iodide (PI) solution	Sigma-Aldrich	Cat# P4864
Hoechst-33342, bisBenzimide H 33342 trihydrochloride	Sigma-Aldrich	Cat# B2261
Hoechst-33258, bisBenzimide H 33258 trihydrochloride	Sigma-Aldrich	Cat# 861405
Dimethyl sulfoxide (DMSO)	Avantor	Cat# AMREN182
Alpelisib (BYL719)	Selleck Chemicals	Cat# S2814
Alpelisib	MedChemExpress	Cat# HY-15244
Bortezomib	Selleck Chemicals	Cat# PS-341
Glycine	Sigma-Aldrich	Cat# G7126
Bovine serum albumin (BSA)	Sigma-Aldrich	Cat# AG3059
Formaldehyde solution (FA)	Sigma-Aldrich	Cat# 252549
Triton X-100	Sigma-Aldrich	Cat# T9284
Tween 20	Sigma-Aldrich	Cat# 274348
Cultrex Reduced Growth Factor Basement Membrane Extract, type 2 (BME-2)	R&D Systems and Amsbio	Cat# 3533-010-02

**Software and algorithms**		

MetaXpress high-content image acquisition and analysis software	Molecular Devices	https://www.moleculardevices.com/products/cellular-imaging-systems/acquisition-and-analysis-software/metaxpress
ImageXpress Confocal HT.ai	Molecular Devices	https://www.moleculardevices.com/products/cellular-imaging-systems#High-Content-Imaging
Adobe Illustrator	Adobe	https://www.adobe.com/dk/products/illustrator. html?gclid=CjwKCAiAzp6 eBhByEiwA_gGq5BmWf OlMLRqS-u9PY8XLn5QXj19Qjzrt2Ff HEVScPHu8jEr3hTyNMR oCLeYQAvD_BwE&mv=s earch&mv=search&sdid= KCJMVLF6&ef_id=CjwKC AiAzp6eBhByEiwA_gGq5 BmWfOlMLRqS- u9PY8XLn5QXj19Qjzrt2Ff HEVScPHu8jEr3hTyNMR oCLeYQAvD_BwE:G:s&s_ kwcid=AL!3085!3!596531663185!e!!g!!illustrator!1480122684!60147185994
BioRender	BioRender	https://www.biorender.com/

**Other**		

384-well, black/clear, tissue culture-treated, Falcon, flat bottom with lid	Falcon	Cat# 353962
Screenstar, 96-well microplate, black, 190 mm clear bottom, cell culture-treated	Greiner	Cat# 655866

## Materials and equipment

**Table:** PDO experimental media

Reagent	Final concentration	Amount
Plasmax or HPLM		239 mL
HEPES	10 mM	5 mL
Primocin	1:100-diluted	500 μL
50 x B-27 Supplement (minus vitamin A)	1:50-dilution	5 mL
SB202190	0.5 μM	12.5 μL
A83-01	0.5 μM	25 μL
Recombinant human FGF-10	10 ng/mL	25 μL
Recombinant human FGF-4	10 ng/mL	25 μL
β-Estradiol	100 nM	25 μL
2-hydroxybutyric acid	0.06 μM	1 mL
**Total**	**N/A**	**250 mL**

Store at 4°C for up to 2 weeks.

***Note:*** The composition of this media is developed for ovarian cancer. The optimal growth factor composition needs to be determined if a different type of cancer organoids is used.

### Live/dead staining solution

• add 4 μL Hoechst 33342 Trihydrochloride Trihydrate (Hoechst 33342) and 4 μL propidium iodide.

(PI) in 1 mL PBS.

Prepare fresh every time.

### Immunofluorescent staining: Fixation buffer

16% formaldehyde solution (FA)/PBS: add 4.3 mL PFA 37% in 5.7 mL PBS.

Prepare fresh every time.

### Immunofluorescent staining: Quenching buffer

• 0.2 M glycine/PBS: add 1.5 *g* glycine in 100 mL PBS.

Store at 4°C for up to 1 month.

### Immunofluorescent staining: Permeabilization buffer

• 0.5% Triton X-100/PBS: add 500 μL Triton X-100 in PBS.

Store at 4°C for up to 1 month.

### Immunofluorescent staining: Washing buffer

• 3% BSA /PBS: add 3 *g* BSA in 100 mL PBS.

Store at 4°C for up to 1 month.

**Table:** Immunofluorescent staining: Blocking buffer.

Reagent	Final concentration	Amount
BSA	1%	3 μg
Triton X-100	0.1%	100 μL
Tween	0.05%	50 μL
PBS	N/A	99.45 mL
**Total**	**N/A**	**100 mL**

Store at 4°C for up to 1 month.

## Step-by-step method details

### Processing and seeding of PDOs in imaging plates for functional assays

**Timing: 2 h followed by 3–4 days incubation**

This major step describes the process of PDOs dissociation from the BME-2 domes where they are cultured, PDOs dilution in fresh BME-2 and finally, PDOs seeding in imaging plates for further functional assays to be performed.

***Note:*** PDOs are used for drug treatment experiments when they reach a stable growth rate and are proliferating and healthy, approximately in passage 10. Organoid size and growth rate varies greatly between different patient derived cultures. Therefore, the size and the organoid density prior to processing is dependent on the growth dynamics of the individual PDO model and should be evaluated carefully. PDOs ready for experiments at approximately 90% confluency are seen in Figure 1.

### Harvesting of HGSC PDOs

1. Aspirate culture media from BME-2 organoid cultures.
2. Wash each well once with 1 mL PBS at 15°C–25°C. Discard PBS.
3. Add 2 mL TrypLE Express in each well at 15°C–25°C.
  a. Incubate for 1–2 min in the tissue culture hood.
4. Detach the BME-2 organoids droplets from the plate bottom with a cell scraper.
  a. Use a sterile single-use cell scraper for each sample.
5. Dissociate the BME-2 organoids droplets by vigorous pipetting 7–8 times until you have a homogenous solution. While pipetting, rinse the whole well with the TrypLE Express solution.

**CRITICAL:** Pre-coat the pipette tip with TrypLE Express for this step. Otherwise, organoids might stick to the plastic rims of the pipette tip, and you may lose material.

6. Incubate the dissociated BME-2 organoid droplets in the plate with TrypLE Express for 15 min at 37°C for the BME-2 gel structure be dissolved.

***Note:*** Seeding densities should be tested and adjusted based on the individual PDO. As a starting point, we suggest that 1 well from a 6-well plate with 10 BME droplets of 20 μL each grown to 90% confluency is suitable for seeding 20 wells of 20 μL each in a 96-well plate and 50 wells of 10 μL each in a 384-well plate.

7. Swirl the plate with the organoids to collect the sample in the middle of the well and transfer the organoid suspension to a 15 mL conical tube.
  a. First, aspirate some microliters of liquid and then collect the organoid-suspension.

**CRITICAL:** Pre-coat the pipette tip with TrypLE Express for this step. Otherwise, organoids might stick to the plastic rims of the pipette tip, and you may lose material.

8. Spin the organoids down by centrifuging them at 240 × *g* for 5 min at 15°C–25°C.

### Seeding of HGSC PDOs in imaging plates

9. Gently aspirate the supernatant.
10. Re-suspend the organoid pellet into cold BME-2 dilution by carefully pipetting up and down without creating any air bubbles and until reaching a homogenous solution.
  a. Dilute the BME-2 with PBS in a 50:50 ratio to seed in the 384-well plates.
  b. Dilute the BME-2 with PBS in a 70:30 ratio to seed in the 96-well plates.

***Note:*** We suggest that for the 96-well plates, the assay is performed in triplicates and in the 384-well plates, the assay is performed in quadruplicate. This should be considered when seeding.

11. Seed PDOs in imaging plates.
  a. Seeding in 384-well plates:
    i. Use a multichannel pipette and seed 8 μL of the diluted BME-2 suspension to each well.
    ii. When depositing the suspension, ensure that the pipette tip touches the bottom of the well.
    iii. Complete the seeding and tap the plate to even the spreading of the BME-2- organoid suspension spreads evenly into the wells.
      ***Note:*** Use the multichannel pipette with a maximum of 10 μL for precise small volumes. Multichannel pipettes with larger volumes also create more bubbles when seeding.
  b. Seeding in 96-well plates:
    i. Seed 20 μL of the diluted BME-2 suspension to the middle of each well.
    ii. Add the pipette tip to the middle at the bottom of the well and create a drop of 20 μL.
    iii. If the matrix does not cover the whole well, gently tap the side of the plate to distribute the matrix.
12. Incubate the plate for 45 min at 37°C to allow complete BME-2 solidification.
13. Add pre-warmed experimental media on top of the wells by avoiding touching the bottom of the plate.
  a. Add 50 μL of PDO experimental media (human plasma mimicking medium such as HPLM or Plasmax with indicated supplements) in the 384-well plates.
  b. Add 100 μL of experimental media (HPLM or Plasmax with indicated supplements) in the 96-well plates.

***Note:*** Pre-heat the appropriate volume of experimental media while incubating at step 12.

***Note:*** To minimize evaporation from the wells, add PBS in the same amount as experimental media in all wells in the rim of the plate.

**CRITICAL:** Deposit the experimental media with slow speed; otherwise, the BME-2 structure might be disrupted by mechanical force.

**Figure 2:**
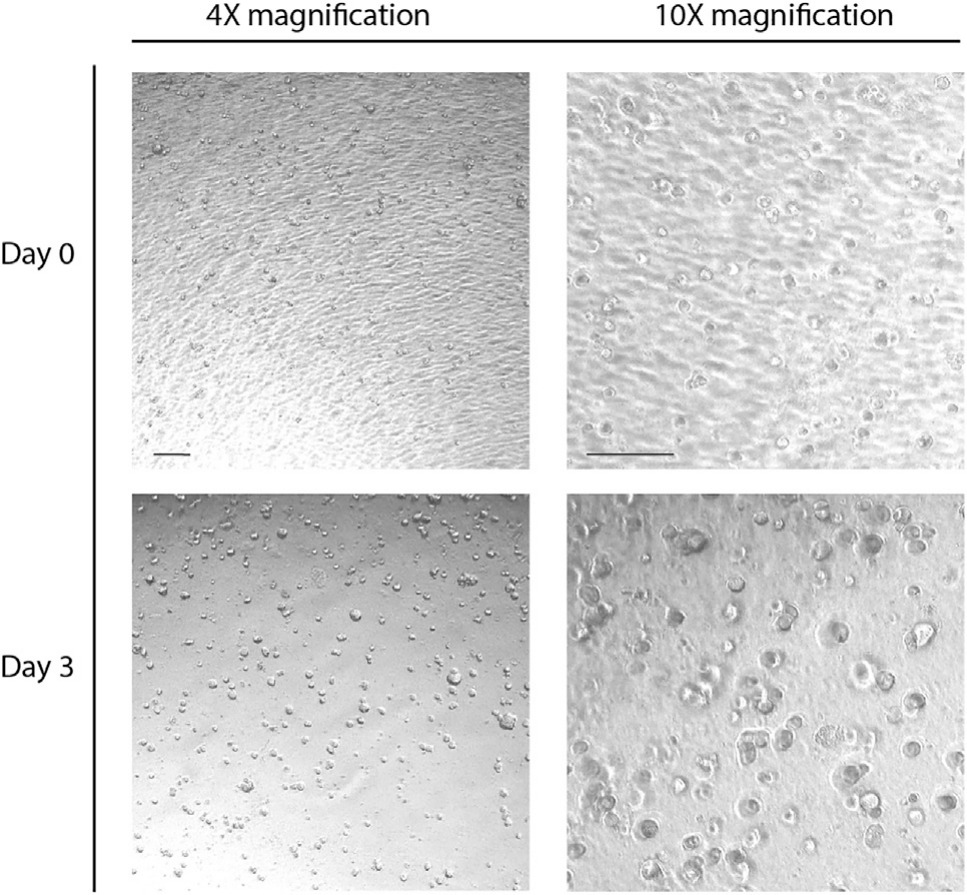
Growth of organoids imaged just after seeding and after 3 days of incubation Images taken with 4X and 10X magnification. Scale bar is 400 μm.

14. Incubate organoids for 3–4 days at 37°C until the PDOs reach a suitable size and density. Example of growth over 3 days in 96-well plates is seen in Figure 2.

**CRITICAL:** To avoid the BME-2 to solidify to early, fast working and having both BME-2 and PBS cooled on ice are necessary. Always pre-cool the pipette tip in cold PBS before aspirating BME-2 solution.

### Drug treatment of PDOs

**Timing: 1 h**

This step describes how to perform a desired drug treatment in PDOs seeded in imaging plates.

15. Prepare the 2x drug dilutions in experimental media.
16. Add the 2x drug dilution on top of the PDOs in the same volume of media that was added per well in step 13.
  a. Include DMSO as a negative control treatment to indicate the amount of spontaneous, drug-independent cell death. The percentage of DMSO dilution should be equivalent to the highest concentration of the drug dilution that has been used in the experiment.
  b. Include 10 μM Bortezomib in the cytotoxicity assay as a positive control treatment to indicate maximum cell death.
17. Incubate treated PDOs at 37°C for desired time. In our experiments with PI3K-inhibitors we used:
  a. 48 h for Ki67 antibody staining.
  b. 72 h for cytotoxicity assay.

***Note:*** To ensure an accurate 2x drug volume to be added in step 16, include three control wells with experimental media when seeding in step 13. Before applying the drug treatment, measure the average amount of evaporation from the control wells. Then, add the measured evaporated volume of experimental medium to the wells with PDOs to regain the initial volume. Then add the 2x drug solution to the PDOs.

***Note:*** Bortezomib is often used as a positive cell death control since it can induce cell death in various solid tumors including ovarian, colon, prostate, breast, and colorectal cancer.^17^^,^^18^ However, its efficacy as a positive control should be tested in case the protocol is used in PDOs deriving from a different type of cancer.

***Note:*** To measure cytotoxicity of PDOs continue with Staining 1 (step 22). To detect and quantify proliferation proceed with Staining 2 (step 33).

### Staining 1: Cytotoxicity assay

**Timing: 2.5 h in total**

This major step describes how to stain for live and dead cells in PDOs seeded in imaging plates and how to measure cytotoxicity via high-throughput confocal screening.

***Note:*** The assay described below refers to PDOs seeded in 384-well plates. The same steps could be used to assay PDOs seeded in 96-well plates by adjusting the volumes of the added compounds.

### Label total amount of cells and dead cells

**Timing: ∼ 1 h**

18. Dilute 4 μL Hoechst 33342 Trihydrochloride Trihydrate (Hoechst 33342) and 4 μL propidium iodide (PI) per 1 mL experimental media to stain and evaluate the total number of cells and the total number of dead cells, respectively.
19. Add 10 μL of the staining solution on top of the PDOs.
20. Incubate for 45 min at 37°C.

### Image acquisition

**Timing: ∼ 1 h**

***Note:*** Our imaging protocols presented below have been set up for ImageXpress HT.ai (Molecular Devices). Other high-throughput microscopes can be used when setting the conditions suitable for them.

21. Image with ImageXpress HT.ai at 10X magnification.
  a. Use DAPI channel to identify Hoechst 33342 and Texas Red channel to identify the PI.
  b. Use transmitted light to control PDO vitality.
  c. Choose a desired number of areas or sites (e.g., 3–4) to be imaged per well.
  d. Use the DAPI channel to adjust the focus and set the optimal exposure for Texas Red to avoid image saturation.
  e. Perform scanning of each well with z-stack acquisition of 5–10 μm step size. Choose the number of z-stacks to ensure scanning of the whole PDO volume. Save both z- stack images and the 2D projection image.
  f. Use the same settings throughout the scanning of the whole plate.

### Image-based analysis pipeline

**Timing: ∼ 30 min**

22. In the custom module workflow at the MetaXpress analysis software, select the ‘Find Round Objects’ function and based on the DAPI image, find all the Hoechst 33342–positive nuclei as individual round objects. Identify them as all nuclei indicating the total number of cells (both live and dead cells) as seen in Figure 3A.

**Figure 3:**
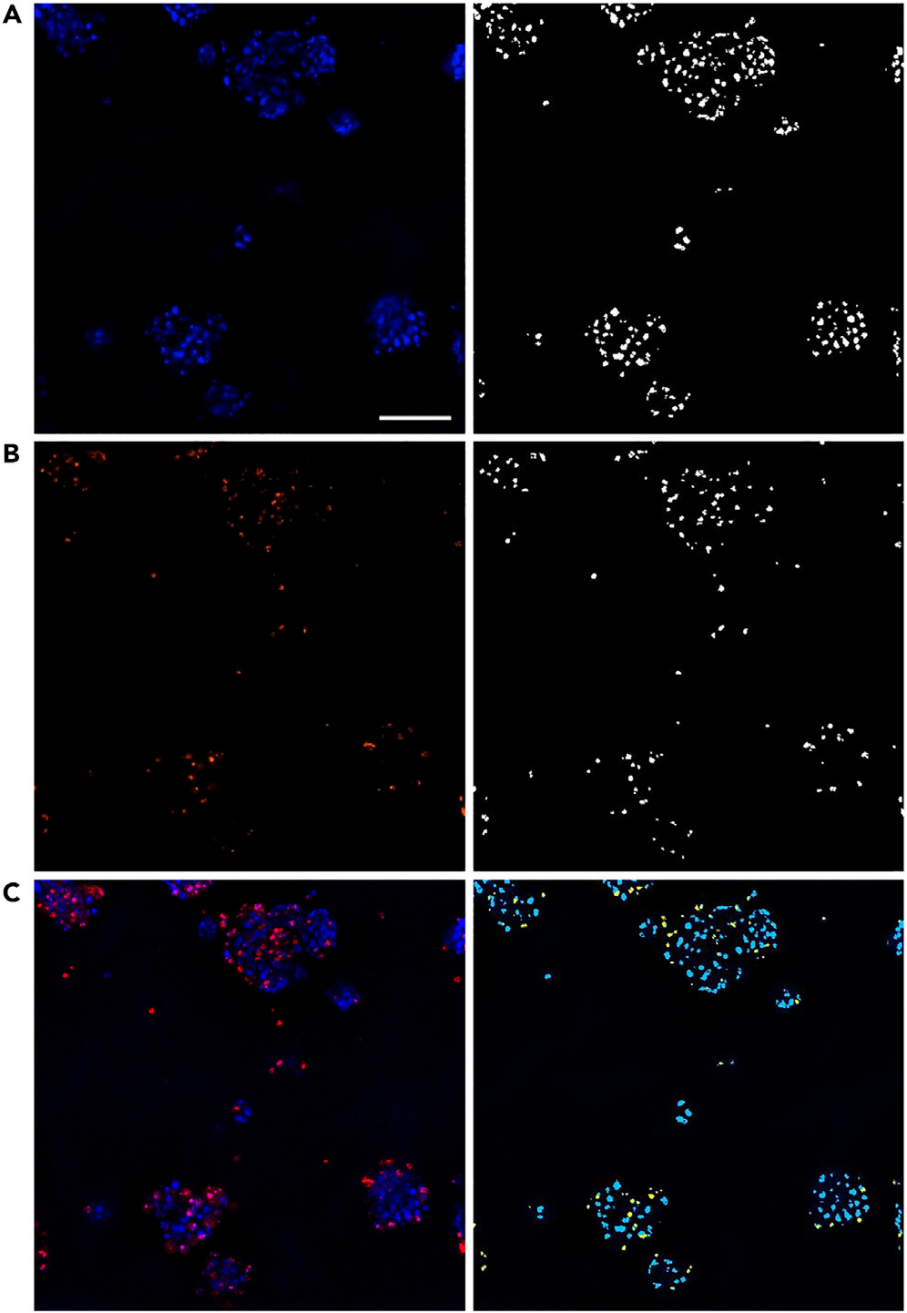
Overview of total/dead MetaXpress analysis pipeline (A) Defining the total number of cells based on the DAPI image (Hoechst 33342 staining). (B) Defining the number of dead cells based on the Texas Red image (PI incorporation). (C) Mask of objects to measure and objects within the mask. Total number of cells depicted as blue and dead cells depicted as yellow. Scale bar is 140 μm.
23. Similarly, by selecting the “Find Round Objects” function and based on the Texas Red image, find all the PI-positive nuclei as individual round objects as seen in Figure 3B. The cells with red nucleus are designated as dead cells.
24. With the function ‘Keep marked objects’, identify the PI-positive red nuclei that overlap with the Hoechst 33342-stained blue nuclei and identify them as the total number of dead cells.
25. Set as the “Mask of objects to measure” to be all nuclei in the DAPI image and set as ‘Features within each object’ to be the dead cells in the Texas Red image as seen in Figure 3C.
26. Set the output of the measurement as “Count of objects”.
27. Export the image-based measurements as sum calculations per site.

### Quantification

**Timing:** ∼**15 min**

28. Calculate the percentage of dead cells out of total number of cells in each condition. Estimate the death index by normalizing to the mean of the negative control (set up as 100% viability) and mean of the positive control (set up as 0% viability).

**Figure 4:**
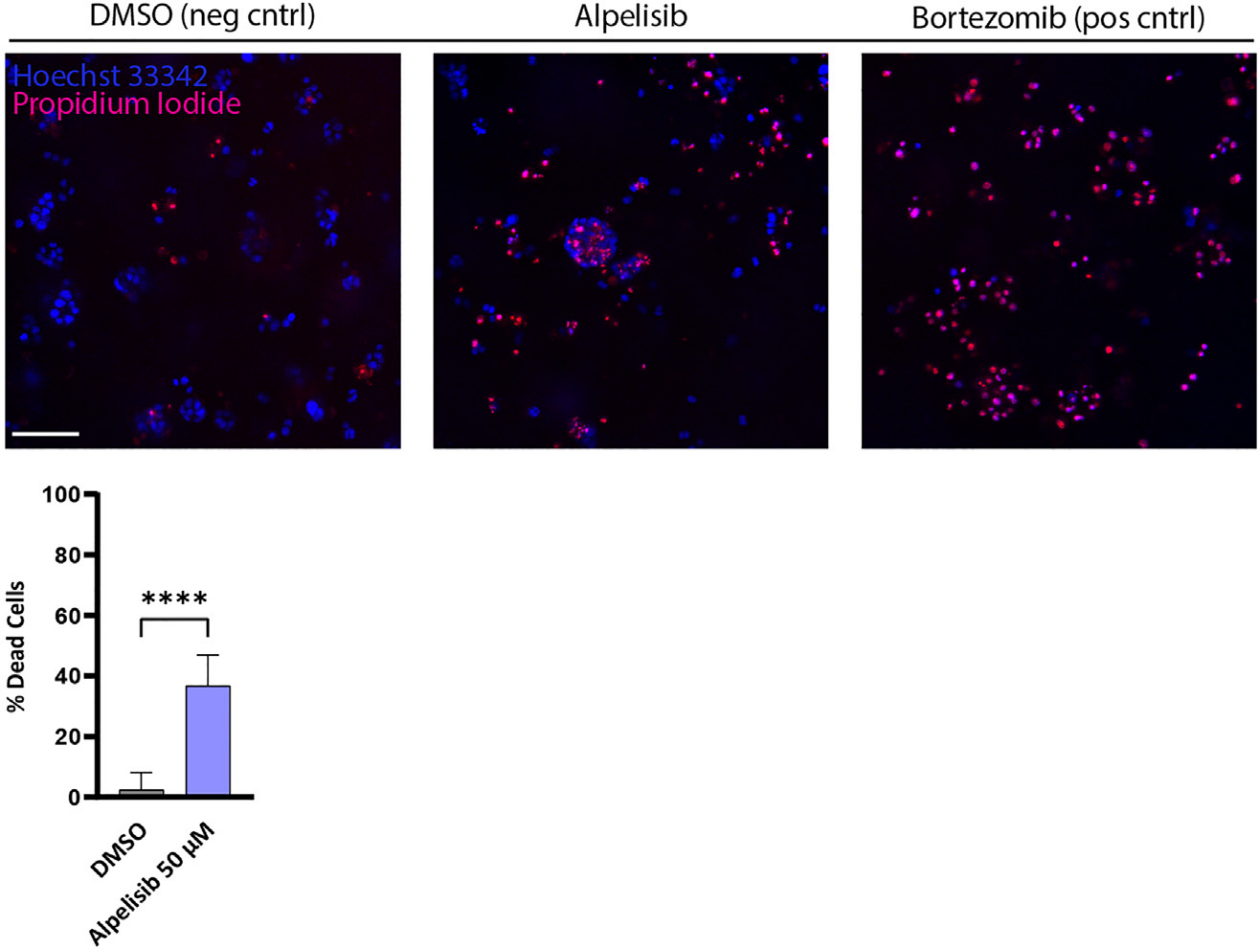
Cytotoxicity assay PDOs labeling with Hoechst 33342 and PI to define total number of cell nuclei and dead cells, respectively, after 72 h drug treatment. DMSO is used as a negative control for cell death, bortezomib (10 μM) is used as a positive control treatment to define cell death, alpelisib (50 μM) is the drug treatment to be validated. Scale bar is 100 μm. Example of quantification of cytotoxicity assay shown with a bar graph representing the mean ± SD. ∗ = p ≤ 0.05, ∗∗ = p ≤ 0.01, ∗∗∗ = p ≤ 0.001 and ∗∗∗∗ = p ≤ 0.0001.

Example of the negative control, drug treatment and positive control for cell death of PDOs is depicted in Figure 4.

### Staining 2: Ki67 antibody staining

**Timing: 2 days in total**

This major step describes how to perform immunofluorescent Ki67 labeling of PDOs seeded in imaging plates and, thus, how to access proliferation of PDOs via high-throughput confocal screening. Continue from step 21.

***Note:*** The assay described below refers to PDOs seeded in 96-well plates. The same steps could be used to assay PDOs seeded in 384-well plates by adjusting the volumes of the added compounds.

### Immunofluorescent staining day 1

**Timing: ∼ 3 h**

***Note:*** All incubations and washes are done with gentle shaking of the plate at 15°C–25°C unless otherwise specified.

29. Remove 100 μL media from the wells to only have 100 μL left covering the organoids.
30. Add 35 μL fixation buffer on top of the 100 μL media in the wells for a final concentration of 4% FA and incubate for 15 min.
31. Wash 3 x with 100 μL PBS for 5 min to remove the FA.

**Pause point:** This step might be used as a pause point. Preserve the plate after by covering it with parafilm and store at 4°C for up to 2 months.

32. Add 100 μL quenching buffer and incubate for 15 min to quench aldehyde groups to decrease the background.
33. Wash 3 x with 100 μL washing buffer for 10 min to prevent non-specific binding.
34. Add 100 μL permeabilization buffer for 15 min to permeabilize the organoids.
35. Wash 3 x with 100 μL washing buffer for 10 min.
36. Add 100 μL blocking buffer and incubate for 1–2 h.
37. Add 70 μL primary antibody diluted in the blocking buffer and incubate the plate for 16–24 h at 4°C with gentle shaking.
  a. Ki67 (mouse) diluted 1:150.
  b. E-cadherin (rat) diluted 1:250.

### Immunofluorescent staining day 2

**Timing: ∼ 3 h**

38. Wash 3 x with 100 μL blocking buffer for 10 min to remove the excess antibody.
39. Add 70 μL of secondary antibody diluted in blocking buffer and incubate for 2 h.
  a. Donkey anti-mouse (Alexa Fluor 594).
  b. Goat anti-rat (Alexa Fluor 488).
40. Wash 3 x with 100 μL blocking buffer for 10 min to remove excess antibodies.
41. Wash once with 100 μL PBS for 5 min.
42. Add 70 μL Hoechst-33258 diluted 1:1000 in PBS and incubate for 30 min to stain the nuclei.
43. Wash 3 x with 100 μL PBS for 10 min.
44. Add 200 μL PBS for imaging.

**CRITICAL:** Do not remove the liquid completely while staining. Leave 10–15 μL covering the BME-2- embedded PDOs. Never let the pipette tip touch the bottom of the well, this will damage and remove the BME-2.

***Note:*** At least 200 μL liquid per well is needed for imaging to avoid disturbing the laser auto focus.

**Pause point:** This step might be used as a pause point. Preserve the plate by covering it with parafilm and tinfoil and store at 4°C for up to 3 months.

### Image acquisition

**Timing:** ∼**2 h**

45. Image with ImageXpress HT.ai with 40X magnification and water immersion lens with the wavelengths for DAPI, FITC, Texas Red and transmitted light.
  a. Use DAPI channel to identify Hoechst 33258, Texas Red to identify the Ki67 and FITC to identify the E-cadherin.
  b. Transmitted light is used for control purposes but is not relevant for the quantification.
  c. Choose 8–9 sites to be scanned per well.
  d. Perform scanning of each well with z-stack acquisition of 1 μm step size. Choose the number of z-stacks to ensure scanning of the whole 3D analyzed BME-2-organoid volume. Save both z-stack images and the 2D projection image.

### Image-based analysis pipeline

**Timing:** ∼**45 min**

46. In the custom module workflow of the MetaXpress analysis software select the “Cell Scoring Objects” function and based on the DAPI and FITC image, find all nuclei with a surrounding cytoplasmic marker around them as seen in Figure 5A.

**Figure 5:**
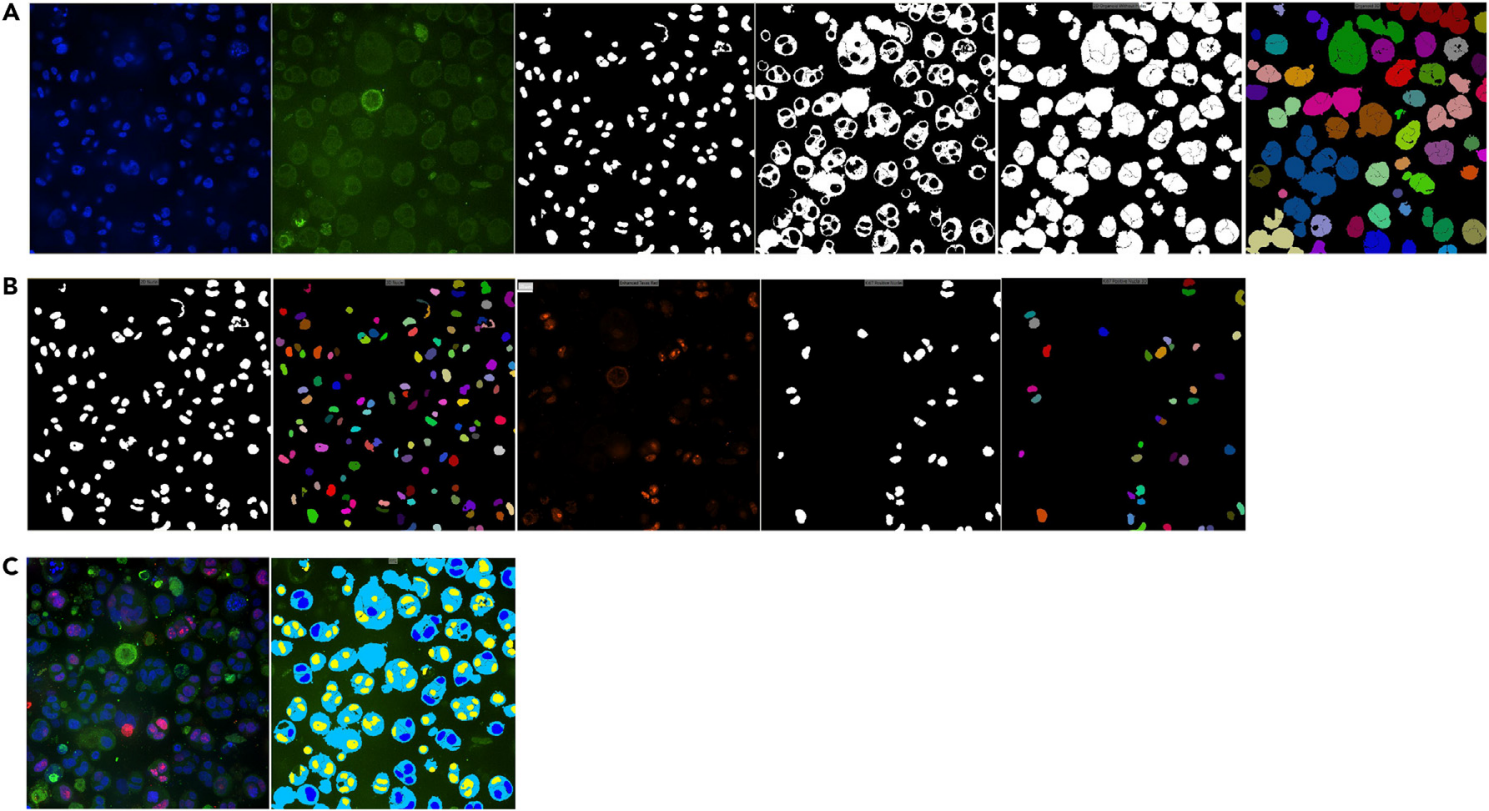
Overview of Ki67 MetaXpress analysis (A) Defining the nucleus and cytoplasmic markers to collect these for the organoid mask, which then is converted into a 3D mask. (B) Making the nucleus mask into a 3D mask and defining which nuclei are positive for Ki67 and making a 3D mask for this. (C) Comparison between the 2D projection image and the mask of a z-stack from the same image. Scale bar 20 μm.
47. Use the 3D function “Connect by touching” to overlay the “cell scoring objects”-mask through all z-stacks to identify the organoids as seen in Figure 5A.
48. Use the mask of the nuclei found in “cell scoring objects” and apply the 3D function “connect by best match” to define the nuclei in 3D as seen in Figure 5B.
49. Use the function “filter mask” to filter the nuclei positive for Ki67 by setting a threshold for the Texas red intensity within the nuclei as seen in Figure 5B.
50. Use the mask of the Ki67 positive nuclei found in filter mask” and apply the 3D function “connect by best match” to define the Ki67 positive nuclei in 3D as seen in Figure 5B.
51. In the measurement pane set the “Mask of objects to measure” to be the 3D organoids within the FITC or DAPI image and set two “Features within each object”. First, the 3D nuclei within the DAPI picture and then 3D Ki67 positive nuclei within the Texas Red image the combined masks can be seen in Figure 5C.
52. Set the output of the measurement to be “Count of objects”.
53. Export the image-based measurements as mean of calculations per site.

### Quantification

**Timing:** ∼**15 min**

54. Calculate the percentage of Ki67 positive nuclei out of total number of nuclei per condition. Estimate the amount of proliferation by normalizing to the mean of the negative control (set up as 0% proliferation) and mean of the positive control (set up as 100% proliferation) control values.

**Figure 6:**
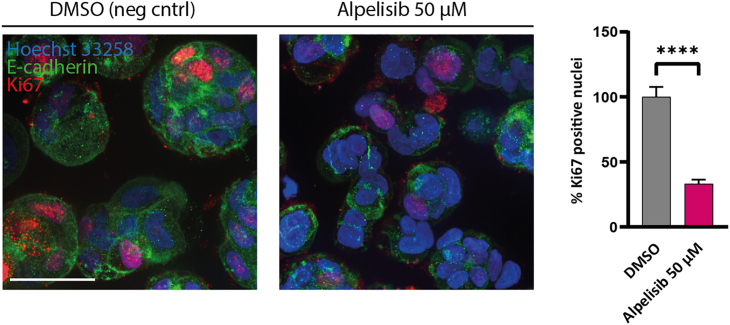
Ki67 antibody staining PDOs staining with Hoechst 33258, E-cadherin and Ki67 to define total number of nuclei and nuclei positive for Ki67 after 48 h drug treatment. DMSO is used as a negative control and PI3K-inhibitor alpelisib (50 μM) as the drug treatment to be validated. Scale bar is 20 μm. Example of quantification of proliferation shown with a bar graph representing the mean ± SD. ∗ = p ≤ 0.05, ∗∗ = p ≤ 0.01, ∗∗∗ = p ≤ 0.001 and ∗∗∗∗ = p ≤ 0.0001.

Example of the negative control and drug treatment of PDOs stained with proliferation marker Ki67 is depicted in Figure 6.

***Alternative:*** The image analysis can also be done by only defining the nuclei and scoring these positive or negative for Ki67. E-cadherin is used to define and visualize the organoids, but not in the quantification, so it can be left out of the analysis.

## Expected outcomes

This protocol allows for rapid drug screening of PDOs assessed by cytotoxicity assay and/or proliferation assay via 3D high-throughput microscopy. When the organoids are established, multiple drugs and drug combinations in multiple patient samples can be tested in parallel as the setup with 384-well plates allows a minimal use of material. Drug efficiency on organoids is validated via measuring cell death and/or proliferation ability after 48 or 72 h treatment using different drug concentrations. Additional time points should be tested for different types of drugs when setting up the assay. If a drug intervention has been suggested by genomic profiling of specific organoids, presented methods can bridge and validate bioinformatics results in an *ex vivo* setting.

## Limitations

PDOs grow in sample-dependent rate and size. Organoids derived from different patient or even different tissue type might need longer incubation times when seeding for experiments and this must be identified carefully in advance.

To ensure biological and statistical significance, PDOs’ density and size should be consistent between experimental repeats. PDOs’ passage should not differ a lot among the repeats, as mutational changes might occur when culturing organoids for a long time. Otherwise, mutational status should be verified among the different passages via DNA sequencing. In addition, since density of PDOs might affect drug potency, apart from being consistent with the density one could also verify by pilot experiments the proper density the drug should be used with.

Although PDOs have translational capacity is in precision medicine, one should take into consideration the absence of stromal components and immune cells of the tumor microenvironment. Since drug response might be affected crucially by those factors, the next step application of these protocols should be on *ex vivo* co-culture systems with tumor associated cells such as fibroblasts and macrophages.

## Troubleshooting

### Problem 1

Air bubble formation while handling BME. It might be caused either by incomplete thawing of BME-2 or by fierce pipetting (related to Step 14).

### Potential solution

Ensure to always thaw the BME-2 gradually on fresh ice. Additionally, make sure to pipette slowly. In case of air bubbles from pipetting, leave the BME-2 on ice for several minutes until the air bubbles move to the top of the mixture and eventually break.

### Problem 2

No formation of organoids after seeding or sparse organoid seeding (related to Step 18).

### Potential solution

Organoid formation may vary among samples due to their intrinsic growth capacity. Culture organoids for longer time in imaging plates to adjust this difference before drug treatment. Exchange the medium every 3 days.

### Problem 3

Weak DAPI signal in cytotoxicity staining (related to Step 22).

### Potential solution

In case of a weak DAPI signal, incubate the PDOs in Hoechst-33342 solution for 1–3 h. Then, incubate for additional 10 min with the PI.

### Problem 4

High background signal in Ki67-staining (related to Steps 42 and 44).

### Potential solution

Ensure proper washing with the blocking buffer containing Tween 20 and Triton X-100 after antibody incubation. Increase incubation time with the washing solution up to 15 min and increase washing steps up to five times.

## Resource availability

### Lead contact

Further information and requests for resources and reagents should be directed to and will be fulfilled by the lead contact, Tuula Kallunki (tk@cancer.dk).

### Materials availability

This study did not generate new unique reagents.

### Data and code availability

This study did not generate new datasets or code.
